# Beyond the Tropics: Unraveling the Complex Relationship between Sun Exposure, Dietary Intake, and Vitamin D Deficiency in Coastal Malaysians

**DOI:** 10.3390/nu16060830

**Published:** 2024-03-14

**Authors:** Shameena Gill, Alia Maisara Adenan, Emillia Elza Thomas, Arifah Haleelur Rahman, Noor Baitee A. Rahim, Noor Akmal Shareela Ismail

**Affiliations:** Department of Biochemistry, Faculty of Medicine, Universiti Kebangsaan Malaysia, Jalan Yaacob Latif, Bandar Tun Razak, Kuala Lumpur 56000, Malaysia; a178344@siswa.ukm.edu.my (S.G.); a176990@siswa.ukm.edu.my (A.M.A.); a174762@siswa.ukm.edu.my (E.E.T.); a176989@siswa.ukm.edu.my (A.H.R.); baitee.rahim@ukm.edu.my (N.B.A.R.)

**Keywords:** vitamin D deficiency, dried blood spots (DBS), skin tones, gender, coastal population

## Abstract

Despite Malaysia’s year-round sunny climate, vitamin D deficiency is surprisingly common among Malaysians. However, we hypothesise that vitamin D levels among coastal populations are above average. Thus, we aim to investigate vitamin D levels and correlate them with the potential contributing factors from three selected coastal villages in Johor, Melaka, and Negeri Sembilan. Convenient sampling was employed to recruit 120 Malay male and female participants, and dried blood spots (DBS) were obtained to measure 25 (OH) vitamin D3 levels via immunoassay. Participants also completed two questionnaires: the Sun Exposure and Protection Index (SEPI) and a validated food frequency questionnaire for Malaysians. The participant pool comprised 35.20% males and 64.80% females who completed all questionnaires and underwent DBS sampling. Our analysis revealed a significant difference (*p* < 0.05) based on skin tones, impacting various facets of the SEPI, including sunscreen usage, protective clothing utilisation, and the adoption of protective headwear. Furthermore, gender emerged as another pivotal factor, demonstrating significant distinctions in these SEPI components. Nevertheless, there is a weak correlation between SEPI scores and vitamin D levels. Subsequent regression analysis did produce statistically significant results (*p* = 0.018), yet the associated low R^2^ value indicated a weak correlation between dietary vitamin D intake that impacts vitamin D levels. In conclusion, our preliminary findings indicate that sun exposure and dietary factors are not the sole determinants of 25-OH vitamin D3 levels. However, we require more samples from various coastal locations for a definitive justification.

## 1. Introduction

Vitamin D, also known as calciferol, is essential for normal bone development and maintenance by enhancing the absorption of calcium, magnesium, and phosphate [1]. It can be obtained from various sources, including sunlight, food, and dietary supplements. Sun exposure enables vitamin D synthesis, with approximately 50% to 90% absorbed through the skin [1]. This process involves multiple steps, beginning with 7-dehydrocholesterol in the skin and through exposure to ultraviolet B rays [2].

Determining optimal vitamin D concentrations remains challenging, but it is generally agreed that levels below 30 nmol/L (12 ng/mL) are considered deficient [3]. Vitamin D deficiency can result from various factors, such as reduced sun exposure [4], low dietary intake of foods containing vitamin D [5], skin colour, clothing choices, and malabsorption syndromes like coeliac disease [6], inflammatory bowel disease [7], short bowel syndrome [8] and venous thromboembolisms [9]. Such deficiencies can lead to chronic hypocalcemia [10] and hyperparathyroidism [11], increasing the risk of osteoporosis, falls, fractures, particularly in older adults, and rickets in children [12].

Malaysia, located near the equator, maintains a tropical climate characterized by steady warmth and humidity throughout the year. In contrast to Western nations, which have four distinct seasons, Malaysia encounters a monsoon season, bringing heavy rainfall from November to March, and a dry season, marked by reduced precipitation, from June to September. Due to its equatorial position, Malaysia experiences a relatively consistent number of daylight hours year-round. Surprisingly, despite Malaysia’s equatorial location with abundant sunlight, local studies have revealed a significant prevalence of vitamin D deficiency among Malaysians [2,13]. This discrepancy is noteworthy given the country’s tropical climate, which receives an ample 7–8 h of intense daily sunlight, far exceeding the 6–8 min required for adequate vitamin D production, as stated in a previous Nordic study [14].

Assessing vitamin D status in plasma is commonly performed using 25-hydroxyvitamin D3 (25-OH vitamin D3), formed in the liver from vitamin D3 [15]. Dried blood spot (DBS) sampling, a cost-effective and minimally invasive method used in various mass screenings for diseases like malaria [16] and HIV [17], pharmacokinetic studies [18], and several metabolic studies [19], is increasingly favoured for its practicality, minimal blood requirement, stability over time, and low risk of contamination [20]. However, its effectiveness in screening vitamin D levels warrants further study.

Coastal areas of Malaysia have not been extensively studied regarding 25-OH vitamin D3 levels, creating a research gap for these specific populations. Therefore, this study aims to investigate differences in 25-OH vitamin D3 levels among coastal dwellers, shedding light on the factors influencing these levels.

## 2. Methodology

### 2.1. Research Design

**Figure 1:**
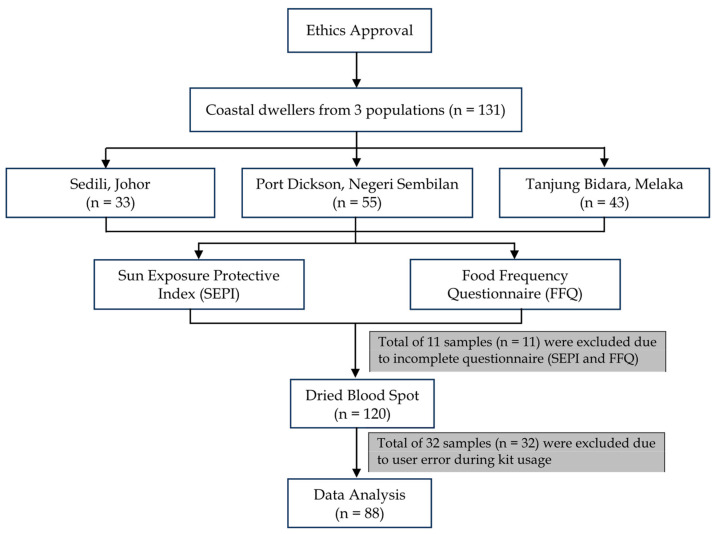
Flowchart of the overview of research design.

This research was an exploratory, cross-sectional pilot study involving the coastal dwellers in Malaysia, namely Sedili, Johor; Tanjung Bidara, Melaka; and Port Dickson, Negeri Sembilan. A targeted and convenient sampling approach was used in this research to maximise the efficacy in recruiting as many young and older adults of Malay ethnicity coastal dwellers as possible with priority to their availability and willingness to participate. A quantitative cross-sectional research design was deemed appropriate to study the 25-OH vitamin D3 status across three (3) different populations of coastal dwellers through laboratory biochemical analyses (DBS and EUROIMMUN 25-OH vitamin D ELISA). A total of 131 participants were recruited for this pilot study across three locations. However, as shown in Figure 1, only 88 complete samples were finally analysed due to incomplete questionnaires and errors during the analysis process.

#### 2.1.1. Subject Recruitment

A few villages were selected based on their geographical location in the predetermined areas to ensure that they fit the population criteria. Permission was then obtained from the head of the village (“Ketua Kampung”) for us to carry out data collection. Free health screening was offered where the villagers underwent a routine health screening, including measuring blood pressure and glucose levels. Convenient sampling was the main frame used to recruit participants for this pilot research to obtain dried blood samples after health screening in the villages. Before obtaining the dried blood samples, an information sheet was provided to inform the purpose of the study and its benefits. Having read and understood the information sheet and informed consent form, the respondents’ signatures serve as their agreement and acknowledgement of voluntary participation.

Villagers of voluntary participation were subjected to a finger prick to check for their blood glucose as routine screening. Then, the remaining blood drops were taken and transferred to the Whatman filter paper; any excess blood drops obtained and not used in this research were discarded. The collected DBS samples were dried in a shaded area with still air. Once the samples were dry, as evidenced by the colour change of the blood spots, the filter paper was packaged in individual airtight plastic containers with desiccant in each. These samples were then transported back to the lab and stored in a −80 °C freezer until further use. A quantitative cross-sectional research design was deemed appropriate to study the 25-OH vitamin D3 status across three (3) different populations of coastal dwellers through laboratory biochemical analyses (DBS and EUROIMMUN 25-OH vitamin D ELISA). A total of 131 participants were recruited for this pilot study across three locations.

The inclusion criteria for the participants are those of Malay ethnicity and those who have reached the legal definition of adulthood, which is outlined as 18 years of age in the Age of Majority Act 1971. Participants were excluded if they had diseases known to affect 25-OH vitamin D3 status, including chronic liver disease, chronic renal disease, and hypo- or hyperthyroidism. Participants who did not complete any part of the questionnaires were also excluded from the final tally.

#### 2.1.2. Biochemical Analysis of DBS

The samples were then analysed with the EUROIMMUN ELISA test kit (Euroimmun, Lübeck, Germany). In the first analysis step, the calibrators and patient samples were diluted with biotin-labelled 25-OH vitamin D3 and added to microplate wells coated with monoclonal 25-OH vitamin D3 antibodies. During the incubation, an unknown amount of 25-OH vitamin D3 in the patient sample and a known amount of biotin-labelled 25-OH vitamin D3 compete for the antibody binding sites in the microplate wells plate. Unbound 25-OH vitamin D3 is removed by washing. To detect bound biotin-labelled 25-OH vitamin D, a second incubation is performed using peroxidase-labelled streptavidin. In a third incubation using the peroxidase substrate tetramethylbenzidine (TMB), the bound peroxidase promotes a colour reaction. The colour intensity is inversely proportional to the 25-OH vitamin D concentration in the sample [21]. Results for the samples were calculated directly using a standard curve.

#### 2.1.3. Questionnaire

A questionnaire was used to study the correlation between 25-OH vitamin D3 levels with diet and sunlight exposure among coastal dwellers. Respondents had to declare demographic information, which was collected to gain background information on the participants of the study. As this study focused on 25-OH vitamin D3 levels, pregnancy and lactation statuses were necessary to be declared under demographic data as participants were separated into different groups. The questionnaire included a section adopted from a validated questionnaire in “Risk factors of vitamin D deficiency among 15-year-old adolescents participating in the Malaysian Health and Adolescents Longitudinal Research Team Study (MyHeARTs)” [22]. This section was used to obtain information on participants’ sunlight exposure based on the Fitzpatrick scale skin pigmentation, duration under the sun, timing of outdoor activities, and the surface area of the body exposed to sunlight. Permission had been successfully obtained from Quah et al. (2018) [22] to use their developed questionnaire that had been validated for the local population to achieve the research objective. A section on sun habits and sun protective behaviours was also included in the questionnaire through a readily available scoring assessment Sun Exposure and Protection Index (SEPI), which allowed the quantitative measurement of current sun behaviours and the inclination to improve sun protection. A food frequency questionnaire (FFQ) was another section of the questionnaire that measured the dietary intake of 25-OH vitamin D3 foods for this study. It was adapted from a semi-quantitative food frequency questionnaire (FFQ) with a strong and positive Spearman correlation coefficient (r = 0.810), which was also the first validated FFQ for 25-OH vitamin D3 intake in Malaysia. This FFQ also included supplement and fortified food intake to account for certain groups of the population who regularly consume these foods. This information was collected in the form of Microsoft Excel sheets (Version 2021). Permission to use this FFQ in this study had been successfully obtained from Zaleha et al. (2015) [23].

### 2.2. Data Collection

#### 2.2.1. DBS and Data Processing

DBS samples and questionnaire data collections were obtained from three different coastal populations of rural areas across Malaysia: Sedili, Johor; Tanjung Bidara, Melaka; and Port Dickson, Negeri Sembilan. These populations were necessary to create a representation of the 25-OH vitamin D3 status in rural areas of Peninsula Malaysia, as different locations contributed to different geographical factors affecting 25-OH vitamin D3 levels. The questionnaire for this study was conducted by creating a data collection form for systematic data collection. This form consisted of four parts: sociodemographic profile, sunlight exposure, sun exposure habits, and the propensity of respondents to increase sun protection. The data collection form was generated online via a Google Form platform (https://docs.google.com/forms/d/e/1FAIpQLScotWWdhyhZkH405LAird25AVEvvgP-0zumv-cOCxQHORPR4Q/viewform, accessed on 1 August 2022), while FFQ data were obtained through one-to-one interviews, and the responses were entered manually into a Microsoft Excel spreadsheet. The data collected were only accessible to the researchers involved to preserve data confidentiality. Respondents had filled out the data collection form under the guidance of researchers, and the form was designed to allow the respondents to submit only if they had completed each of the questions. This form was then administered to the respondents in the three locations.

#### 2.2.2. The 25-OH Vitamin D3 Status Using DBS

The dried blood samples were then processed and analysed using the EUROIMMUN immunoassay to evaluate the 25-OH vitamin D3 levels of the coastal rural populations through the collection of DBS samples in three separate locations. Detailed biochemical analyses were mentioned in Section 2.1.2.

### 2.3. Statistical Analysis

As shown in Figure 1, out of the 131 samples obtained initially, 11 were excluded from the data analysis due to incomplete questionnaires or FFQs. Out of 120 samples, 88 were analysed using the immunoassay kit due to user error while using the kits. Meanwhile, data from the FFQ were manually calculated using vitamin D values from the United States Department of Agriculture (USDA) Food Database and the Singaporean Health Promotion Board’s Energy and Nutrient Composition Database. This was performed given the presence of multiple data entries that required vitamin D values for foods frequently eaten locally that were unavailable on the USDA database. The information from the databases was standardised amongst the researchers to ensure consistency during the calculation process. 

The differences in responses for SEPI Part 1 (0–32 points) and SEPI Part 2 (0–20 points) between skin types and genders, as well as intake of vitamin D, were also analysed. To investigate statistical differences in median values of the total scores of SEPI Parts 1 and 2, we used the non-parametric independent samples median test; for skin type, we used the Kruskal–Wallis analysis, and for gender, we used the Mann–Whitney *U*-test. Post hoc tests were also carried out to determine specific significant differences. A confidence interval of 95% was set as statistically significant for all analyses. 

Data from the questionnaire were analysed using the IBM Statistical Package for Social Science (SPSS) Statistics 25.0 to determine the correlation between 25-OH vitamin D3 levels with diet and sun exposure among coastal dwellers. After obtaining 25-OH vitamin D3 readings from all DBS and serum samples, the Bland–Altman plot was employed for a graphical finding to see if both sampling methods were within their limits of agreement. To test the associations between 25-OH vitamin D3 readings and demographic data such as gender, skin tone, and SEPI scores, Mann–Whitney’s U-test and independent samples Kruskal–Wallis’s test was carried out, followed by a post hoc Dunn’s test to further scrutinize each of the specific significant differences in the data. Pearson’s chi-squared test was also employed as another statistical test to find the significant difference in 25-OH vitamin D3 categories and demographic data. Spearman’s correlation and logistic regression were also carried out to determine any relationship between 25-OH vitamin D3 levels and vitamin D intake, which was obtained from the FFQ.

## 3. Results and Discussion

### 3.1. Sociodemographic Profile

Initially, a total of 131 participants were enrolled in this study. A total of 88 complete samples were then analysed after excluding incomplete samples and samples destroyed by user error. Of the 88 samples, many of the respondents were female (n = 57), comprising 64.8% of the group, while males constituted 35.2% (n = 31) of the total respondents (Table 1). Almost two-thirds of the respondents (n = 52), 59.1%, were 45 years old and above, with the age range of 55 to 64 (n = 23) having the highest frequency of 26.1%. Close to half of the total participant group (n = 42), or 55.6%, consisted of respondents who were not professionally employed (i.e., housewives, retirees, and unemployed).

Data on the skin tone of the respondents were also collected (Table 2). This was a self-evaluation performed by the villagers and was based on the Fitzpatrick classification of skin tones. A majority of the respondents (n = 25) had type 3 skin. This was followed by type 4 (n = 22), type 2 (n = 21), and type 5 skin (n = 15). Only 5.7% of the population surveyed (n = 5) had type 1, light, and pale white skin, while none of the 88 respondents had type 6 skin. Most of the time spent in the sun was in the early morning (39.4%) and in the late afternoon (27.6%). Only 11.8% of time spent outside was in the early afternoon (11.8%). This is consistent with the temperature changes throughout the day, which rise in the morning and peak in the early afternoon before dipping again in the late afternoon. Additionally, the behaviour of the respondents, especially in terms of clothing patterns, was also asked about in the questionnaire. Four options in terms of body parts exposed to the sun were given for the participants to choose from–“hands and face”, “arms and face”, “arms, face, and legs” as well as “arms, abdomen, face and legs”. The results were evenly split between “hands and face” and “arms and face” with 54.5% of respondents (n = 48) choosing the former and the remaining 45.5% of respondents (n = 40) choosing the latter. This was in line with the conservative style of clothing that is seen in most Muslim Malaysians.

### 3.2. SEPI Score

The SEPI questionnaire was divided into two parts, Part 1 and Part 2. For the former, a higher score indicated an increasing risk of UV exposure, while a higher score for the latter demonstrated a reduced inclination to increase sun protection. The mean and median for both parts of the SEPI questionnaire were calculated along with their *p*-values. Non-parametric tests such as Kruskal–Wallis (skin tone) and Mann–Whitney (gender) were used to obtain the *p*-values for the analysis below.

#### 3.2.1. Sun Habits and Sun Protection Behaviour (SEPI Part 1)

As SEPI Part 1 reflects on increasing UV risk exposure, there were only three components in this section that had significant differences between various skin tones—sunscreen use, protective clothing use, and protective headwear use such as caps and hats. As shown in Table 3, for sunscreen use (*p* = 0.001), respondents with skin tone 5 (brown) had the highest mean (4.00) and median score = 4. Apart from type 1 skin (light and pale white), the mean score for this component increased as the melanin content on the skin increased, with type 2 skin having a mean score of 1.76, followed by type 3 skin with 2.92, and type 4 skin with 3.18. As half of the participants from the group with skin types 3 to 5 (n = 62) never used sunscreen when in the sun (median score = 4), there are respondents with light pale white skin (type 1 skin, n = 5) who seldomly use sunscreen.

For protective clothing use (*p* = 0.005), a similar trend apart from type 1 skin was noted. The highest mean and median for this component were seen in respondents who had type 5 skin, with a mean of 2.27 and a median of 2, respectively, whereby 50% of the brown skin type occasionally wore covering clothes for sun protection. The last statistically significant component was the use of protective headwear (*p* = 0.006), in which an inverse trend was noted. Mean scores decreased from skin type 1 to type 5, alluding that darker-skinned individuals were more likely to wear protective headwear.

The SEPI Part 1 score was also analysed across gender lines to test for any significant differences. Table 4 shows that men had a significantly higher mean and median total score (*p* < 0.01) compared to women, who indicated that men generally had an increased risk of UV exposure due to riskier behaviour when exposed to sunlight. There were significant differences in all three components again—sunscreen use (*p* < 0.001), protective clothing use (*p* < 0.001), and protective headwear use (*p* < 0.05). Men reported a higher mean when comparing sunscreen use and protective clothing use, while women reported higher mean scores in terms of protective headwear use. Based on the median, half of the male participants (n = 31) never use sunscreen, occasionally wear covering clothes for sun protection, and often wear protective headwear. However, half of the women (n = 57) always wore covering clothes and never used protective headwear.

Independent samples of Kruskal–Wallis’s test were then followed up with post hoc Dunn’s test to compare each statistically significant category pairwise. For SEPI Part 1, as reported above, three components were statistically significant—sunscreen use, protective clothing use, and protective headwear use such as caps and hats. For sunscreen use (*p* < 0.001), pairwise comparison indicated that the scores of respondents with skin tone type 2 had significant differences when compared to respondents with skin tone type 5 (*p* < 0.001). This means that in terms of sunscreen use, the group with skin tone type 2 frequently used sunscreens when in direct sunlight compared to those with skin tone type 5. A pairwise comparison for the use of protective clothing showed that the scores of respondents with skin tone type 5 were significantly different from those with skin tones type 2 (*p* < 0.01) and type 3 (*p* < 0.01), indicating that those with skin tone type 5 had a reduced frequency of using covering clothing as protection from the sun compared to the latter skin tones. Respondents with skin tone type 4 were also observed to have a significant difference (*p* < 0.05) in their scores compared to those with skin tone type 2 in terms of using protective headwear. This indicated that those with skin tone type 4 in this group wore caps, hats, and other articles of protective headwear more frequently than those with skin type 2.

#### 3.2.2. Readiness to Increase Sun Protection (SEPI Part 2)

SEPI Part 2 was used to explore the inclination of the respondents to adopt more protective behaviour when out in the sun. The SEPI Part 2 was analysed similarly to the first part, with the mean, median, and total scores calculated along skin tones and gender. Once again, there were significant differences seen in the use of protective clothing (*p* < 0.01), protective headwear (*p* < 0.05), and staying in the shade to avoid direct sunlight (*p* < 0.01) when calculated according to skin tones, as shown in Table 5. For the use of long-sleeved clothing, participants with skin type 4 (olive) had the highest mean with a median of 4, while those with skin type 2 (fair and white) had the lowest mean and median of 0. The medians obtained showed that 50% of olive and fair skin types have never been thought of and have for a long time used long-sleeved clothes for sun protection, respectively. For protective headwear use components, those with skin type 1 had the highest mean with a median score of 0, and skin type 5 had the lowest mean but the highest median score. Participants with skin type 1 were also the least likely to stay in the shade to avoid sunlight, as evidenced by the highest mean score and median of 4. However, participants with olive (type 4) skin are the most likely to stay in the shade during hours of the strongest sunlight, evidenced by the median score of 0 on the 5-point scale.

As seen in Table 6, men had higher mean total scores and were thus less likely than women to change risky behaviours in terms of sun exposure (*p* < 0.05). There were also significant differences in the inclination to wear long-sleeved clothes (*p* < 0.001), wear protective headwear (*p* < 0.001), and stay in the shade (*p* < 0.001) between men and women. Women were more likely than men to start wearing protective clothing and staying in the shade to reduce their exposure to UV. However, men were more likely to don protective headwear than women.

### 3.3. Vitamin D Levels

**Figure 2:**
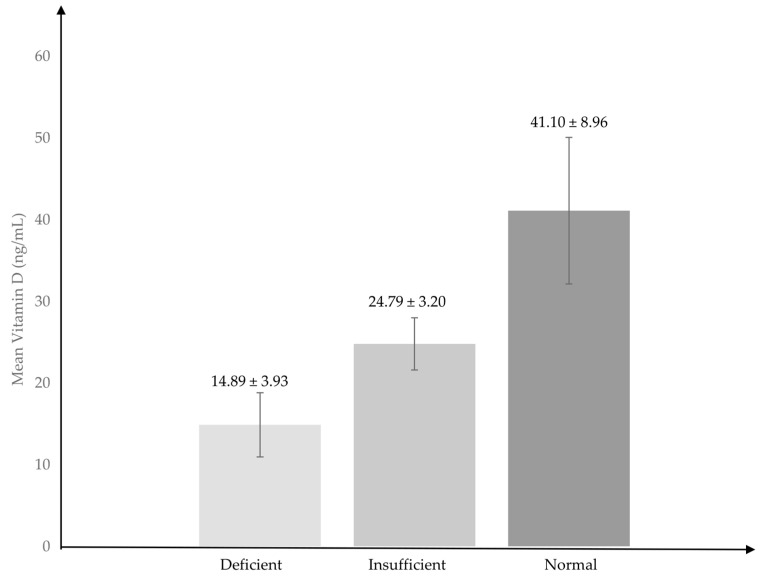
Vitamin D levels and its categories of the Malay coastal dwellers.

Vitamin D levels were characterised into three major groups—insufficient (<20 ng/mL), deficient (20–30), and normal (>30). Two bar graphs were constructed to display the number of respondents in these categories according to gender and skin tone. A majority of the male respondents (n = 11) had deficient levels of vitamin D. There were equal numbers of male respondents (n = 10) who had insufficient and normal levels of vitamin D. Conversely, most of the female respondents (n = 25) had normal levels of vitamin D followed by 19 insufficient and 13 deficient female respondents (Figure 2). A chi-squared analysis of vitamin D level categories and gender showed no statistically significant results (*p* > 0.05).

Respondents with skin tone type 1 were distributed equally among all three vitamin D categories, with two respondents each in the deficient and normal categories and one respondent in the sufficient category. Respondents with skin tone type 3 and type 4 were many respondents with normal levels of vitamin D at 13 and 12 respondents, respectively. Conversely, most of the surveyed population with insufficient levels of vitamin D were those with skin tones type 2 (n = 9), type 3 (n = 7), and type 5 (n = 7). A chi-squared analysis of vitamin D level categories and skin tone also showed no statistically significant results (*p* > 0.05).

**Figure 3:**
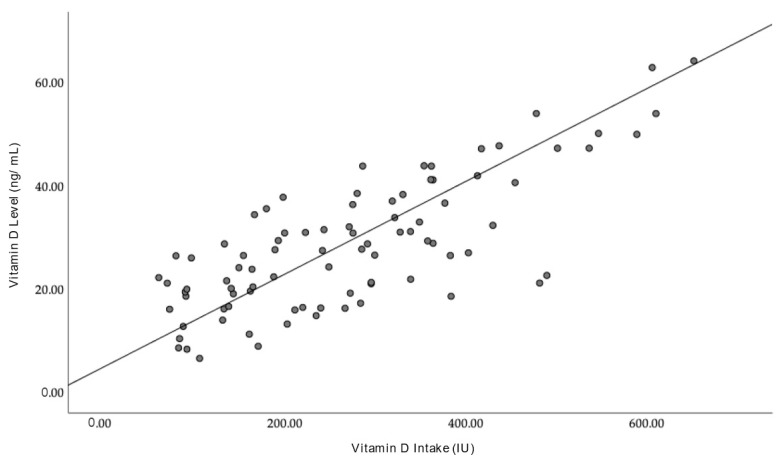
Correlation between vitamin D intake and vitamin D levels of Malay coastal dwellers.

Spearman’s correlation test was performed to test for any relationship between the vitamin D intake of the participants, as calculated from the FFQ, and the subsequent vitamin D levels of the group. There was a positive and linear relationship between dietary intake and vitamin D levels. The regression equation for predicting vitamin D levels from dietary intake is y = 0.09x + 5. The correlation had been statistically significant with r (88) = 0.764 (*p* < 0.001). Despite the statistically significant correlation, dietary intake only predicts 57.9% of the variance in vitamin D levels in this group (Figure 3).

### 3.4. Discussion

#### SEPI Questionnaire

The results obtained indicated that three components in the first part of the SEPI questionnaire were significantly affected by skin tone and gender. Respondents with darker skin tones were generally less likely to use sunscreen and wear protective clothing when out in the sun than those with lighter skin tones. This has already been proven by multiple studies that found that individuals with a lower perceived UV sensitivity, as characterised by the Fitzpatrick skin types, were more likely to incorporate sunscreen usage when exposed to the sun [24,25]. This could be due to a false sense of security or a “sunscreen paradox” rooted in misconception about the amount of melanin in one’s skin and the subsequent protection it offers against UVA and UVB rays [26,27,28,29]. Furthermore, the Fitzpatrick classification for skin types has a poor correlation with UV sensitivity. From a preventive standpoint, the increased frequency of certain practices in the sun in populations with darker skin is not ideal as it places those with perceived low UV sensitivity in danger of overexposing themselves to UV radiation [30,31]. However, the mean scores for the use of protective clothing were lower across the board than sunscreen use, indicating that the group wore protective clothing more often than sunscreen when it came to sun protection, regardless of skin tone type. This could be due to the local demographic makeup of Malaysia, a majority Malay Muslim country. One of the major tenets of the religion is wearing conservative clothing that covers the whole body except the face and dorsum of the hand. Heavy influences from the culture and religion offer defence against the sun’s rays, fit into these characteristics, and thus, could explain the higher frequency of using this method as a form of sun protection. This aligns with findings from a Saudi Arabian study that revealed comparable outcomes, indicating that women tend to wear protective clothing more often, potentially influenced by cultural and religious factors [32]. The above reason could also explain the inverse trend observed for the usage of hats and caps—the frequency of using this method increased as skin type became darker. While this is not in line with previous research, this could be explained by the fact that most Muslim women and some older men already wear religious headwear such as the head covering (hijab, “kopiah” or “songkok”), which would inevitably discourage them from adding on a cap or a hat when going into the sun. However, the effectiveness of these articles of religious headwear against UVA and UV rays has not been studied nor recommended as an alternative for protective headwear use.

Differences between tendencies to adopt safer sun practices were also noted between skin types within the three components, which are (i) staying in the shade, (ii) protective clothing use, and (iii) protective headwear use. Apart from those with skin type 1, those with darker skin types were less likely to adopt safer behaviours in the sun. This could be due to a false sense of security that darker skin helps to protect against UVA and UVB rays and, thus, could hamper individuals from changing their behaviour [26,27,28,29]. However, this trend was reversed when looking at protective headwear use, with the tendency to adopt this behaviour decreasing with darker skin types.

A difference in existing protective habits and an inclination to adopt such habits were also noted between genders. Male respondents had riskier sun habits in terms of low sunscreen usage and protective clothing compared to female respondents. This is similar to existing findings in the literature, which show that men generally endorse riskier sun practices and are more reluctant to implement protective behaviours [33]. However, in this population, female respondents were seen to wear protective headwear less frequently than male participants in this group [33]. This could be due to the pre-existing clothing practices in the community that see Malay Muslim women wear head coverings daily for religious purposes. This may discourage women from wearing sun hats or caps when out in the sun for several reasons, such as discomfort and their understanding that the hijab is adequate for sun protection. In general, men had riskier sun habits, which translated to an increased risk of UV exposure [34].

In terms of inclination to adopt healthier habits, female respondents were less likely to want to stay in the shade or wear long-sleeved clothing when out in the sun. The latter could be explained by the fact that most Malay Muslim women in Malaysia, especially in more conservative rural localities, already wear long-sleeved clothing daily. However, the intention behind this clothing is not for sun protection; rather, it is for religious reasons. This demonstrated the influence of cultural and social norms in determining clothing patterns [35]. From the cultural perspective, this could be because workforce participation in many sectors, particularly outdoor work, is very much influenced by socially constructed gender roles [36]. In this scenario, more men in our group worked jobs that exposed them to regular sun exposure, which may make seeking shade during working hours more difficult than those working indoors. Regardless of this, however, the implementation of wearing long-sleeved clothing has been shown to effectively reduce the risk of sunburn [37]. Conversely, male respondents were less likely to start using protective headwear than their female counterparts. Overall, men were less likely to adopt safer sun habits than women in this study. This concurs with the existing literature that generally groups women as more susceptible to adopting more beneficial habits to reduce over-exposure to the sun, plausibly due to the increased awareness of skin ageing and its after-effect, including cancer [38]. Local studies have shown that while the Malaysian population does have adequate information about safe practices while in the sun, this does not necessarily translate into the incorporation of these behaviours in their daily life [39,40]. Addressing this gap is crucial to ensure that the Malaysian population can enjoy the advantages of sun exposure while also prioritizing safety.

The association between vitamin D levels and other factors such as demographics, exposure to sun, and vitamin D intake was also carried out. Only vitamin D intake yielded a statistically significant finding when compared to vitamin D levels. Regression analysis that was carried out demonstrated that vitamin D intake was significantly associated (R^2^ = 0.764, F(1, 86) = 120.618, *p* < 0.001) with vitamin D intake. This meant that about 57.9% of the variability in the levels of vitamin D could be attributed to dietary intake. This is important in the context of future intervention programmes specific to this demographic. Sun exposure has traditionally been the preferred way of increasing vitamin D levels in tropical countries [41,42,43] due to the abundance of sunlight and the absence of additional cost needed compared to increasing vitamin D intake via supplements or whole foods. However, for a majority of working individuals in Malaysia, enjoying sunlight at the optimum hours of the day (early morning) remains a theoretical concept. Having indoor jobs and commuting early to work means many employed, working-class citizens are unable to reap the benefits of sun exposure as an effective way of increasing vitamin D levels. In this scenario, the above results show that increasing vitamin D intake externally can be a viable alternative to increasing vitamin D levels in a tropical society that is already struggling to reach optimum levels in the general population. The Malaysian population generally has easy access to foods rich in vitamin D in local supermarkets at an affordable price. Being a tropical country near the equator, the UV index is higher than other regions [44]. Although dietary intake is seen as a small contributor to vitamin D synthesis in the body, its effects are not as negligible as the general population assumes [45]. While there is a safe and beneficial limit to sun exposure, prolonged exposure to the sun may predispose the population to other issues, such as skin malignancies [46] and cataracts [47], leading to more health problems in the long run. Therefore, using sunlight as a primary tool to increase vitamin D levels in the general population could be akin to a double-edged sword.

However, these results must be interpreted in the context of the small sample size and niche population that was studied. Further studies could be carried out with a larger group and a more representative population so that these results can be generalised and used for further intervention purposes.

## 4. Strengths and Limitations

The exploration of factors affecting vitamin D levels among coastal Malay dwellers is uncharted territory and, hence, represents a pioneer endeavour. This subject has not been extensively studied before, as most research offers results based on vitamin D levels in the urban population rather than the coastal population. Delving into unknown terrain, this research can offer fresh insights and unique perspectives on this subject. This study stands out as a novel contribution to fill in the landscape where the knowledge gap persists by shedding light on unexplored multifaceted factors of vitamin D dynamics within specific cultural contexts. The distinct focus on coastal Malay dwellers as the sample population adds cultural specificity, acknowledging lifestyle variations and dietary habits that may impact vitamin D levels. The active participation of the coastal Malay dwellers strengthens the research through the incorporation of local perspectives. Coastal regions can have unique environmental factors affecting vitamin D synthesis. The geographical specificity allows the research to be more targeted, and this enhances the research outcome’s applicability to coastal communities in Malaysia. The findings in this research may have implications directly for possible public health policies. Implementing improvements to existing public health policies can be attempted, especially when the research outcome reveals modifiable factors that can be addressed to increase vitamin D levels in the target population. The findings from this study could allow a comparative analysis with other communities as the outcome provides valuable insights into regional variations and contributes to a more comprehensive understanding of the issue. 

The group chosen for this study may limit the use of the research for future studies due to findings not being universally applicable beyond coastal Malay dwellers. The specificity of sample ethnicity limits the potential to generalise results to other populations with different lifestyles, diets, and environmental conditions. Coastal regions, being susceptible to environmental pollution, may have an impact on the quality of sunlight exposure and subsequently affect vitamin D synthesis. While combining quantitative measures such as dried blood spot (DBS) with qualitative data through questionnaires gives a richer dataset as subjective aspects from participants’ lives are also captured, the variability could be a challenge in accurately assessing certain factors influencing vitamin D levels as it relies on retrospective, self-reported data. The reliability of self-reported data and the precision of the measurement tools used may affect the accuracy of the data collected. The participants’ awareness of being part of research focused on vitamin D levels may also influence their behaviour, potentially leading to altered dietary recall or increased sun exposure, hence introducing response bias. The method of collecting dried blood spot samples posed a limitation as Whatman filter paper was utilised instead of a dried blood spot card with pre-printed circles to guide the size of blood spots. This choice led to challenges, specifically an underestimation of blood spot diameters, resulting in the discarding a few samples due to insufficient blood spots for conducting the immunoassay. However, DBS collection still remains a superior way to collect samples, especially in rural populations or places that do not have easy access to laboratories.

## 5. Conclusions

This research provides a valuable perspective for understanding previously understudied facets of public health, specifically focusing on the influential factors affecting vitamin D levels among coastal Malay dwellers. The insights gained from this study can contribute to the scientific discourse on vitamin D dynamics and have significant implications for healthcare strategies.

While Malaysia is a tropical country, it still struggles to address the overarching issue of vitamin D deficiency and its potentially adverse effects on the health of the local population. Through this study, the behaviour of coastal populations in relation to sun exposure can be explored, as well as the factors influencing vitamin D levels in the aforementioned population. These findings can then inform future plans regarding public health policies and messaging to the general population. This, in turn, will enhance health outcomes and help indirectly lower the strain on the public healthcare system, specifically in terms of treating long-term sequelae of chronic vitamin D deficiency. Additionally, this research has shown the multifaceted nature of factors influencing vitamin D levels in the target population. While sunlight is not scarce in Malaysia, a tropical country, vitamin D deficiency remains prevalent, underscoring the need for better health communication strategies and a new approach to effectively address this particular public health concern. The results of our study, while confined to a small population, show that vitamin D supplementation through dietary intake is a step in the right direction, especially when sun exposure habits locally are so heavily influenced by cultural, religious, and societal influences. This could be a valuable tool in handling this hidden epidemic and help lay the foundation for refining health policies and communication strategies.

In conclusion, this research sheds light on the complex interplay of factors influencing vitamin D levels among coastal Malay dwellers, uncovering previously overlooked dimensions in public health and offering a nuanced understanding of the challenges of overcoming vitamin D deficiency in a tropical context. As we move forward, collaborative efforts between researchers, healthcare professionals, and policymakers will be crucial in implementing effective interventions to mitigate the impact of vitamin D deficiency on population health. Furthermore, continued investigation and adaptation of strategies will be essential to ensure sustained improvements in the health outcomes of the coastal Malay population and potentially inform broader public health initiatives in similar regions.

## Figures and Tables

**Table 1:** Sociodemographic profile of coastal dwellers from three villages.

	Frequency	Percentage (%)
**Gender**		
Male	31	35.2
Female	57	64.8
**Total**	**88**	**100.0**
**Age**		
18–24	5	5.7
25–34	12	13.6
35–44	19	21.6
45–54	14	15.9
55–64	23	26.1
65–74	13	14.8
75–84	0	0.00
85–94	2	2.30
**Total**	**88**	**100.0**
**Occupation**		
Administrative	1	1.1
Agriculture and fisheries	4	4.5
Cleaning services	5	5.7
Construction	2	2.3
Driver	1	1.1
Educator	7	8.0
Entrepreneur	14	15.9
Hospitality	5	5.7
Housewife	21	23.9
Manufacturing	1	1.1
Office job	4	4.5
Retired	18	20.5
Shop assistant	2	2.3
Unemployed	3	3.4
**Total**	**88**	**100.0**

**Table 2:** Skin tone of coastal dwellers from three villages.

	Frequency	Percentage (%)
**Skin Tone**		
1.00—Light, pale white	5	5.7
2.00—Fair, white	21	23.9
3.00—Medium to olive	25	28.4
4.00—Olive	22	25.0
5.00—Brown	15	17.0
6.00—Dark brown to black	0	0.0
**Total**	**88**	**100.0**
**Time Spent in the Sun**		
Early morning	50	39.4%
Mid morning	27	21.3%
Early afternoon	15	11.8%
Late afternoon	35	27.6%

**Table 3:** Results of SEPI Part 1 according to skin tone.

	Intentional Tanning	Occasions with Sunburn	Time Spent in the Midday Sun	Sun Vacation Abroad	Sunscreen Use	Protective Clothing Use	Protective Headwear Use	Staying in the Shade	Total Score
Skin Type 1 (n = 5)									
Mean	0.00	0.40	0.40	0.60	2.40	1.00	3.80	1.20	9.80
Median	0	0	0	0	3	0	4	0	11
Skin Type 2 (n = 21)									
Mean	0.33	0.14	0.67	0.38	1.76	0.57	3.14	0.90	7.90
Median	0	0	0	0	0	0	4	1	7
Skin Type 3 (n = 25)									
Mean	0.24	0.20	0.88	0.16	2.92	0.80	2.44	0.92	8.56
Median	0	0	0	0	4	0	4	0	8
Skin Type 4 (n = 22)									
Mean	0.45	0.27	0.91	0.09	3.18	1.23	1.55	0.73	8.41
Median	0	0	0	0	4	0	1	0	8
Skin Type 5 (n = 15)									
Mean	0.00	0.00	1.47	0.27	4.00	2.27	1.67	1.07	10.73
Median	0	0	0	0	4	2	2	0	10
*p*-value	0.379	0.170	0.610	0.292	0.001 *	0.005 *	0.006 *	0.924	0.070

* *p*-value is significant and lesser than *p* < 0.05.

**Table 4:** Results of SEPI Part 1 according to gender.

	Intentional Tanning	Occasions with Sunburn	Time Spent in the Midday Sun	Sun Vacation Abroad	Sunscreen Use	Protective Clothing Use	Protective Headwear Use	Staying in the Shade	Total Score
Female (n = 57)									
Mean	0.14	0.09	0.63	0.23	2.21	0.32	2.71	0.93	7.27
Median	0	0	0	0	2	0	4	0	7
Male (n = 31)									
Mean	0.48	0.35	1.32	0.13	4.00	2.55	1.58	0.77	11.19
Median	0	0	0	0	4	2	1	0	11
*p*-value	0.445	0.465	0.159	0.501	<0.001 ***	<0.001 ***	0.03	0.463	0.001 ***

*** *p*-value is significant and lesser than *p* < 0.001.

**Table 5:** Results of SEPI Part 2 according to skin tone.

	Giving Up Sunbathing	Sunscreen Use	Clothes for Sun Protection	Headwear for Sun Protection	Staying in the Shade	Total Score
Skin Type 1 (n = 5)						
Mean	1.20	2.40	1.00	3.40	0.80	8.80
Median	0.00	3.00	0.00	4.00	0.00	8.00
Skin Type 2 (n = 21)						
Mean	1.95	1.71	0.57	3.24	0.67	8.14
Median	1.00	0.00	0.00	4.00	0.00	8.00
Skin Type 3 (n = 25)						
Mean	1.68	2.68	0.60	2.16	1.00	8.12
Median	0.00	4.00	0.00	4.00	0.00	8.00
Skin Type 4 (n = 22)						
Mean	1.77	3.05	0.73	1.36	0.64	7.55
Median	0.00	4.00	0.00	0.00	0.00	8.00
Skin Type 5 (n = 15)						
Mean	2.13	4.00	1.93	1.40	1.27	10.73
Median	4.00	4.00	2.00	1.00	0.00	10.00
*p*-value	0.884	0.002 *	0.011 *	0.006 *	0.772	0.314

* *p*-value is significant and lesser than *p* < 0.05.

**Table 6:** Results of SEPI Part 2 according to gender.

	Giving Up Sunbathing	Sunscreen Use	Clothes for Sun Protection	Headwear for Sun Protection	Staying in the Shade	Total Score
Female (n = 57)						
Mean	1.75	2.16	0.26	2.72	0.81	7.70
Median	0.00	3.00	0.00	4.00	0.00	8.00
Male (n = 31)						
Mean	1.94	3.84	2.00	1.13	0.97	9.87
Median	2.00	4.00	2.00	0.00	0.00	9.00
*p*-value	0.670	<0.001 ***	<0.001 ***	<0.001 ***	0.555	0.032

*** *p*-value is significant and lesser than *p* < 0.001.

## Data Availability

Data are contained within the article.
